# Gorham-Stout disease affecting the spine with cerebrospinal fluid leakage and Chiari-like tonsillar herniation: a rare case report and review of literature

**DOI:** 10.1186/s12883-023-03092-y

**Published:** 2023-02-03

**Authors:** Qian-qian Xing, Meng Miao, Qiao-wei Zhang, Yue Wu, Fei-fang He

**Affiliations:** 1 Department of Pain Management, Sir Run Run Shaw Hospital, School of Medicine, Zhejiang University, Hangzhou, 310016 Zhejiang People’s Republic of China; 2 Department of Neurology, Qilu Hospital (Qingdao), Cheeloo College of Medicine, Shandong University, Qingdao, Shandong 266035 People’s Republic of China; 3 Department of Radiology, Sir Run Run Shaw Hospital,School of Medicine, Zhejiang University, Hangzhou, 310016 Zhejiang People’s Republic of China; 4 Department of Pain Management, Center for Intracranial Hypotension, Sir Run Run Shaw Hospital, School of Medicine,Zhejiang University, No. 3 Qingchun Road, Hangzhou, 310016 Zhejiang People’s Republic of China

**Keywords:** Gorham-Stout disease, Cerebrospinal fluid leakage, Epidural blood patch, Chiari-like tonsillar herniation, Rebound intracranial hypertension

## Abstract

**Background:**

Gorham-Stout disease (GSD) is a very rare disorder characterized by massive osteolysis of poorly understood aetiology. The association between GSD involving the skull base and cerebrospinal fluid (CSF) leakage has been reported in the literature. However, few cases of CSF leakage and Chiari-like tonsillar herniation in GSD involving the spine have been reported.

**Case presentation:**

We present the case of a 20-year-old man with GSD involving the thoracic and lumbar spine, which caused CSF leakage and Chiari-like tonsillar herniation. The patient underwent four spinal surgeries for osteolytic lesions of the spine over a 10-year period. Here, we discuss the possible aetiology of the development of CSF leakage. Epidural blood patch (EBP) was performed at the T11-T12 level to repair the CSF leakage. After EBP treatment, rebound intracranial hypertension (RIH) developed, and tonsillar herniation disappeared 2 months later.

**Conclusions:**

GSD involving the spine with CSF leakage and Chiari-like tonsillar herniation is relatively rare. For patients who have undergone multiple spinal surgeries, minimally invasive treatment is an alternative treatment for CSF leakage. EBP can repair CSF leakage secondary to GSD and improve chronic brain sagging, with reversibility of Chiari-like malformations.

## Background

Gorham-Stout disease, also known as massive osteolysis, Gorham’s disease, disappearing or vanishing bone disease, idiopathic osteolysis, and phantom bone disease, is a rare disorder of idiopathic and progressive bone resorption, with replacement by lymphatic or vascular and fibrous tissue proliferation. Imaging studies have demonstrated osteolytic lesions without new bone or periosteum reaction, which is significantly different from bone dissolution caused by inflammation or malignancy. The disease was first described by Jackson in 1838; a detailed clinical description was made by Gorham and Stout in 1955 [1]. GSD can involve any part of the skeleton in the body, especially the pelvis, humerus, maxillofacial structures and axial skeleton [2]. The clinical manifestation varies depending on the bone site involved. Spinal involvement is associated with neurological symptoms characterized by scoliosis, CSF leakage or paraplegia. To date, almost 300 cases of GSD have been reported in the literature, of which only approximately 70 cases involve the spine [3]. Due to its rarity and limited studies, the mechanisms underlying this disease are still largely unknown. The clinical course is usually prolonged and can eventually stabilize. Poor prognosis is associated with spine osteolytic lesions, pleural effusion and chylothorax [4].

CSF leakage can cause low intracranial pressure, presenting as postural headache. In some severe cases, brain sagging can develop, resulting in Chiari-like descent of the cerebellar tonsils [5]. Spontaneous CSF leakage is mostly common at the spinal level, especially at the cervical or thoracic spine or cervicothoracic junction [6]. However, CSF leakage in patients with GSD mostly occurs from the osteolytic skull base bone, particularly the temporal bone [7–9]. CSF leakage at the spinal level is relatively rare in osteolysis patients. Here, we present a case of a 20-year-old man diagnosed with GSD with involvement of the spine and secondary CSF leakage.

## Case presentation

**Fig. 1:**
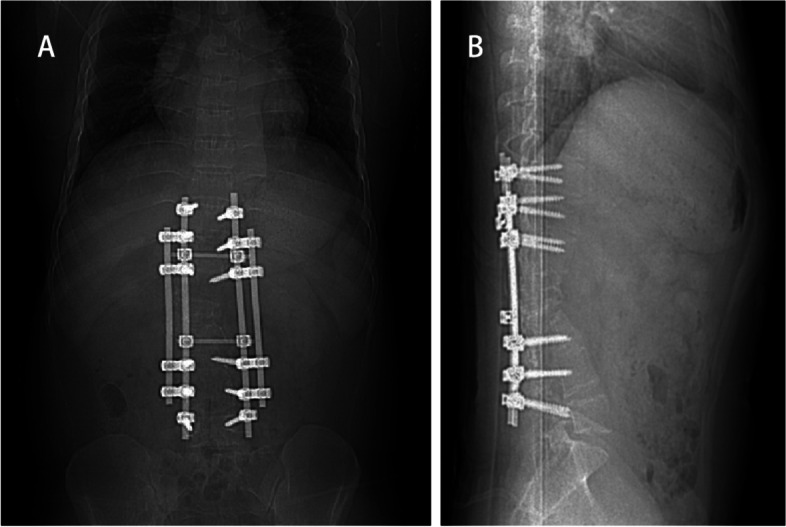
Postoperative anteroposterior (**A**) and lateral (**B**) images indicate an internal fixator shadow at the T10-L5 level

**Fig. 2:**
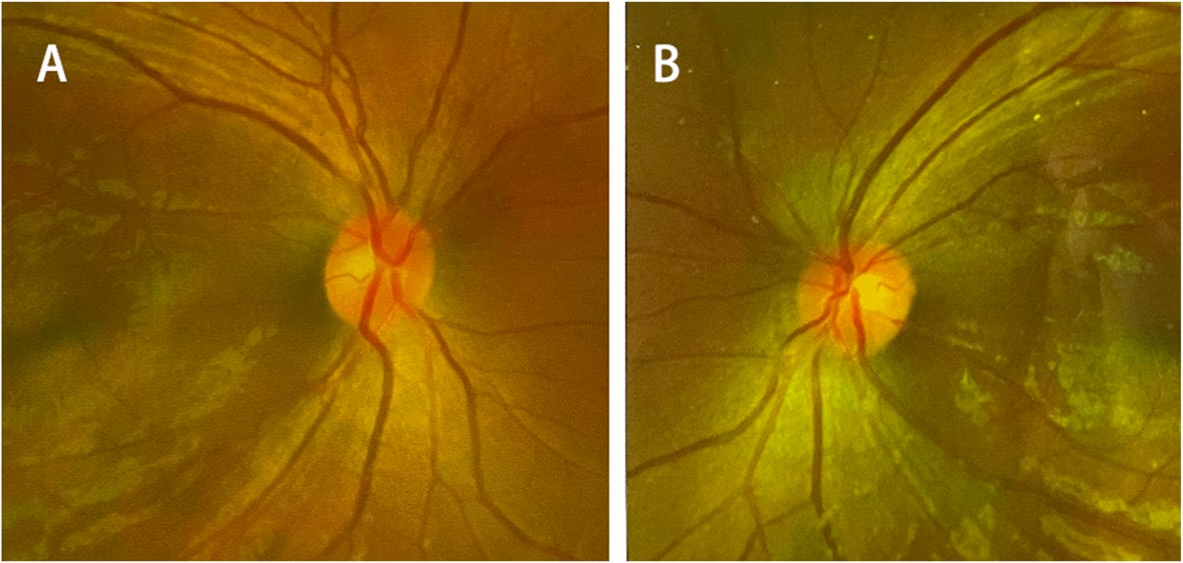
Fundus examination image showing no bilateral papilledema. **A**, Right eye **B**, Left eye

A 20-year-old man suffering from recurrent headache in the standing position for 10 months was admitted to our hospital. He had a history of GSD. In the past twelve years, the patient had undergone a closed thoracic drainage procedure due to chylothorax and 4 spinal surgeries for osteolytic lesions of the spine (Fig. 1). Half a year after the last spinal revision surgery, the patient suddenly developed postural headache, which was aggravated while standing and relieved while supine. He was initially confined to bed at home. Five months prior to admission to our hospital, he was admitted to a hospital in Shan Xi with gradually worsening headache, nausea and vomiting. The patient underwent cerebral angiography, which indicated right sigmoid sinus stenosis. Fundus examination showed no bilateral papilledema (Fig. 2). He was treated with fluid rehydration and oral anticoagulants. The patient’s condition improved.

**Fig. 3:**
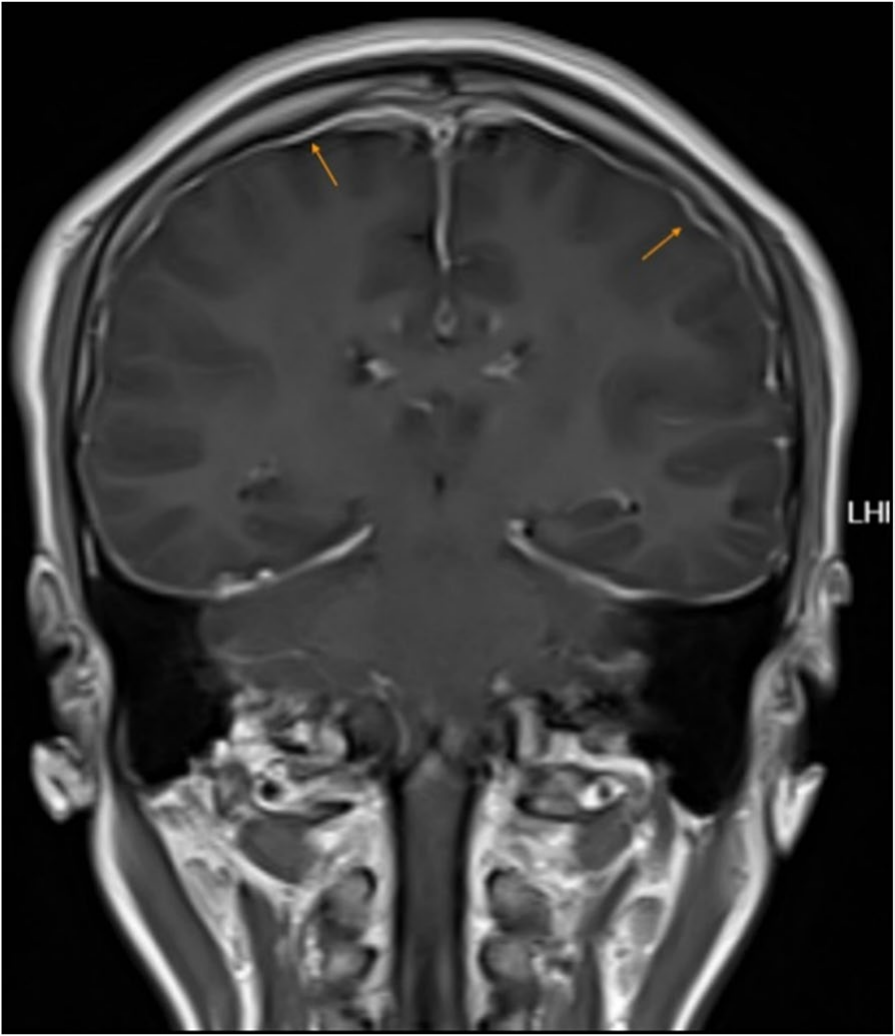
Contrast-enhanced brain MRI demonstrating smooth diffuse pachymeningeal enhancement, which was consistent with intracranial hypotension

**Fig. 4:**
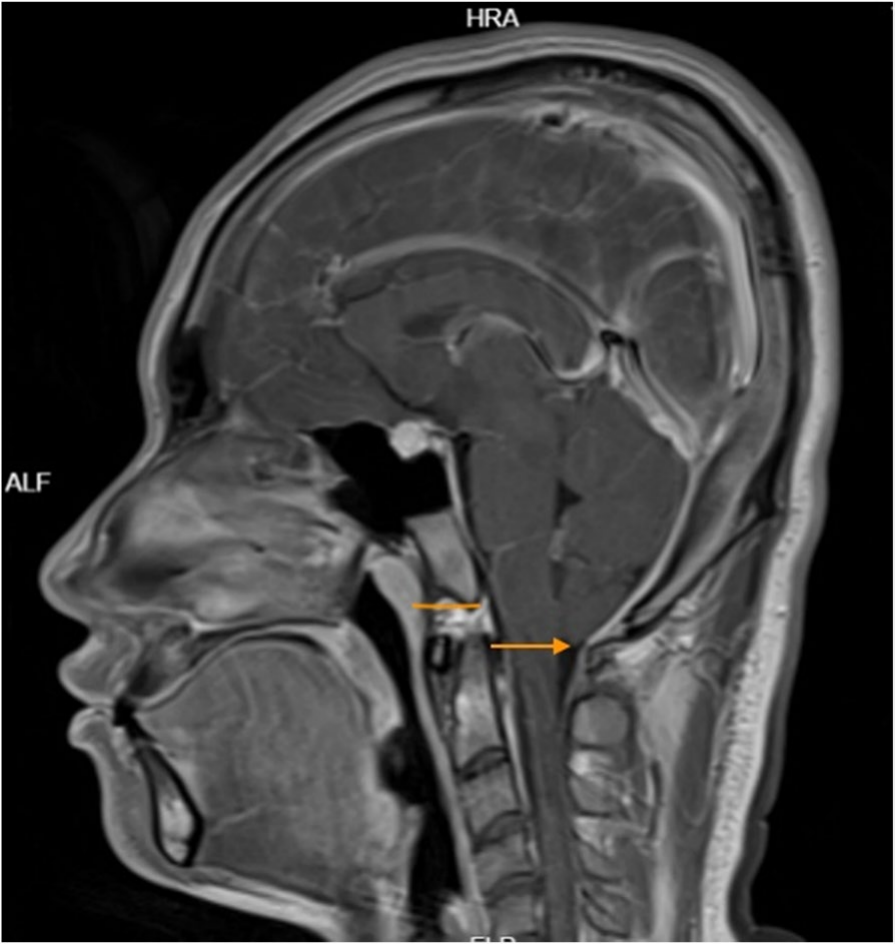
Contrast-enhanced brain MRI demonstrating cerebellar tonsillar herniation

**Fig. 5:**
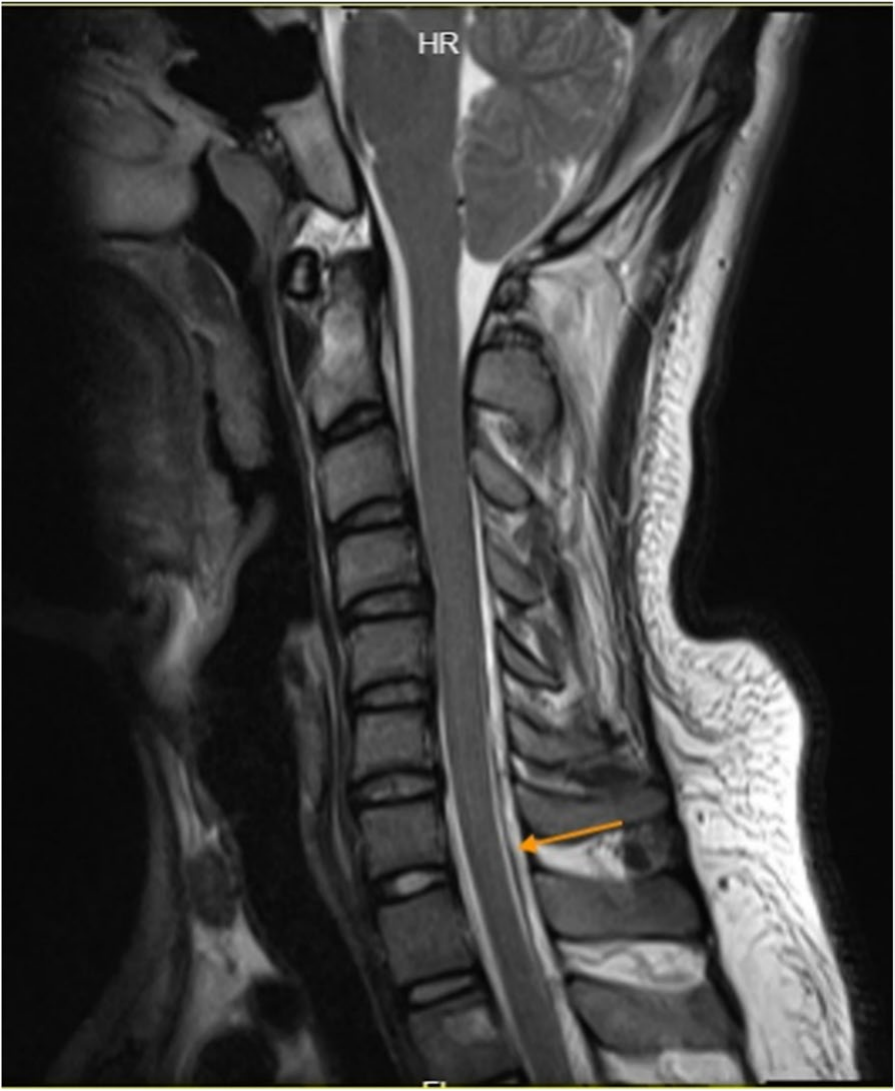
Sagittal T2-weighted MR image of the total spine showing posterior epidural fluid collections in the cervical and upper thoracic segments

**Fig. 6:**
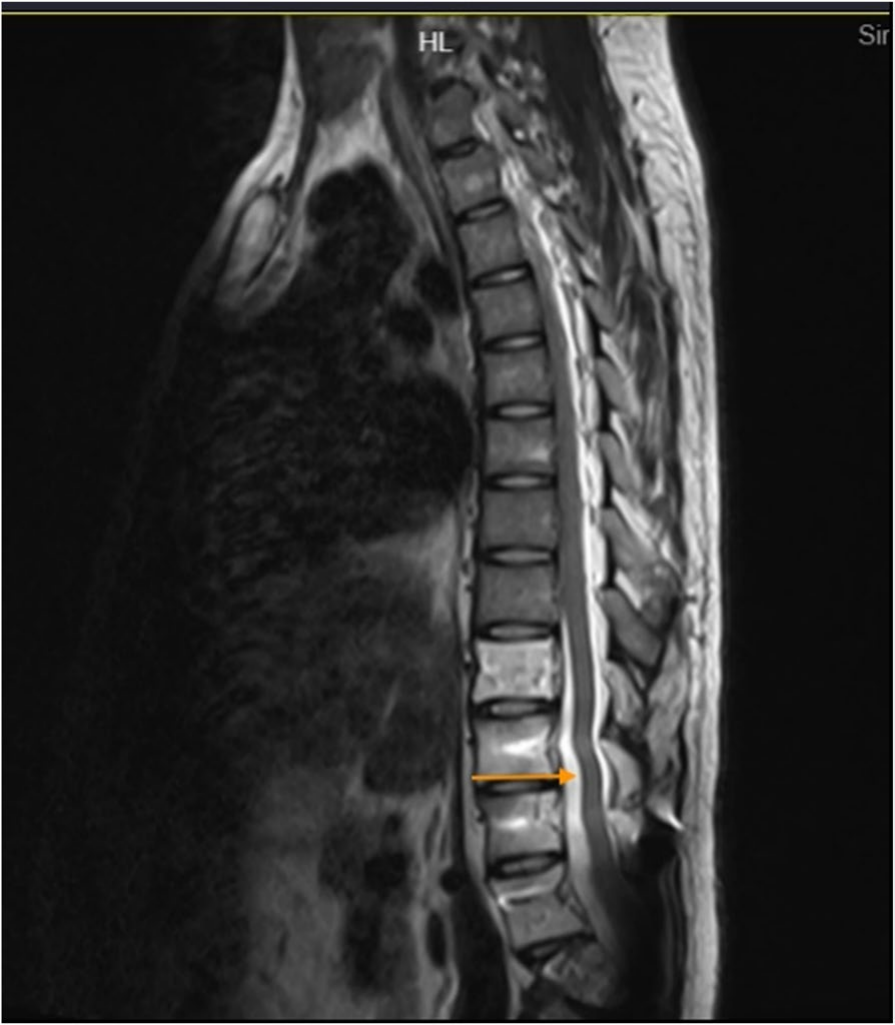
Sagittal T2-weighted MR image of the total spine showing anterior epidural fluid collections in the lower thoracic segments

**Fig. 7:**
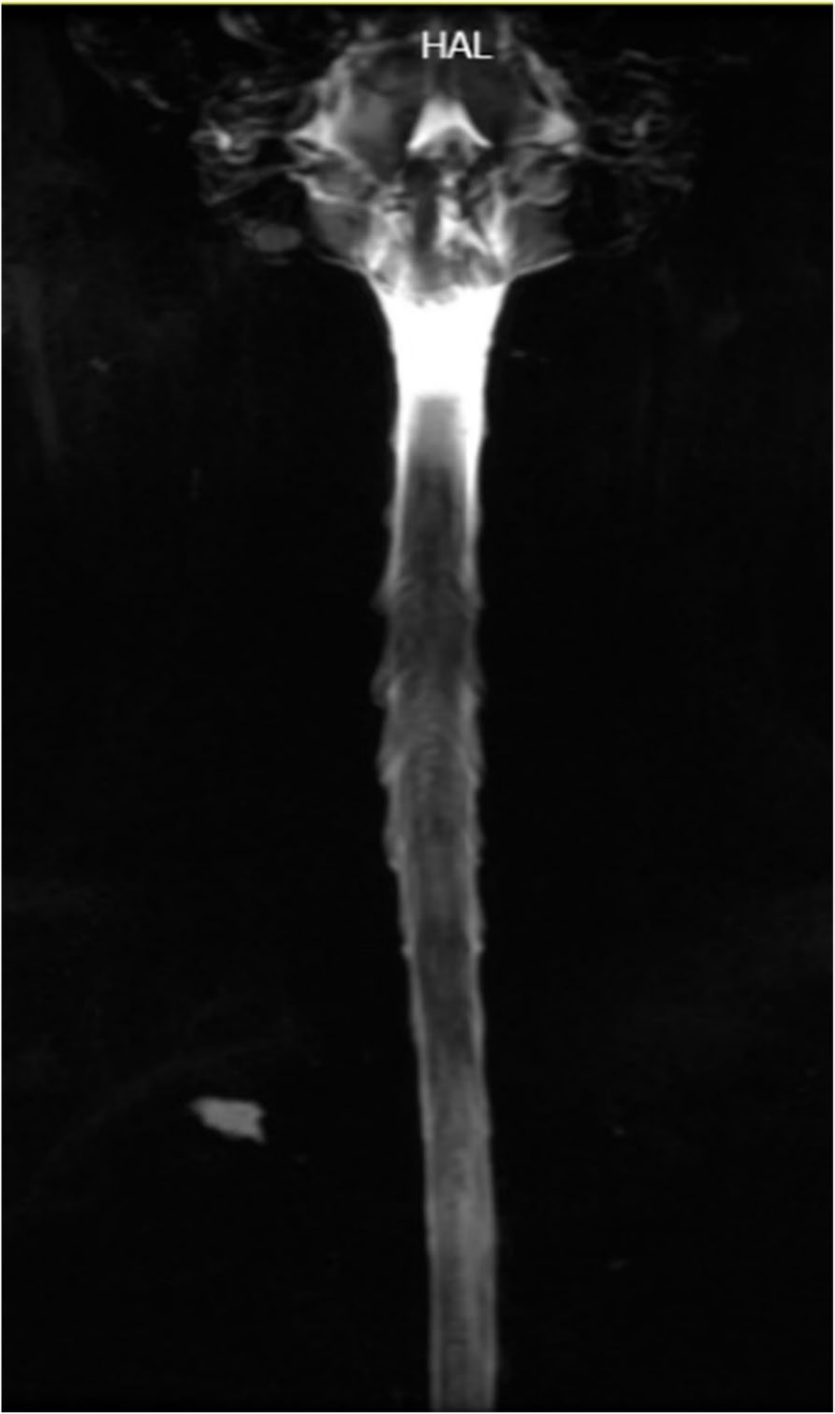
Spine MR myelography image revealing no evidence of cerebrospinal fluid leakage in the cervical and upper thoracic vertebrae

However, the patient could still not stand for a long period of time in recent months. After standing for three hours, the headache worsened and could be relieved by lying on the back. For further diagnosis and treatment, the patient was referred to our hospital. After admission, he was treated with absolute bed rest and intravenous fluid infusion. In the meantime, imaging tests were scheduled for the patient. Contrast-enhanced brain magnetic resonance (MR) images demonstrated smooth diffuse pachymeningeal enhancement and cerebellar tonsillar herniation, which was consistent with intracranial hypotension (Figs. 3 and 4). The sagittal T2-weighted MR image of the total spine showed posterior epidural fluid collections in the cervical and upper thoracic segments and anterior epidural fluid collections in the lower thoracic segments (Figs. 5 and 6). Then, further spinal MR myelography was performed to locate the CSF leak. Spinal MR myelography revealed no evidence of CSF leakage in the cervical and upper thoracic vertebrae (Fig. 7), while images of the lower thoracic and lumbar segments were unclear due to metal artefacts. The clinical and imaging data both indicated intracranial hypotension syndrome, but the current examination methods could not specifically locate the leak, which caused certain confusion for the repair of CSF leakage. Considering the complexity of the case, we organized a multidisciplinary team involving neurologists, neurosurgeons, pain physicians, orthopaedists and radiologists. Subsequently, computed tomography (CT) of the skull base was performed, showing no abnormalities. The lower thoracic and lumbar segments were not visible on MR images due to metal artefacts. Therefore, after ruling out skull base lesions, it was speculated that the possible leakage point was located in the lower thoracic or lumbar segment. Ultimately, we decided to perform lower thoracic epidural blood patching. An 18-G needle was inserted into the patient’s epidural space at the T11/12 level, and 11 mL of autologous peripheral venous blood was infused.

During the next two months, the patient’s headache gradually improved, and he returned to normal activities. However, two months after the EBP, the patient presented to another local hospital with severe headache, orbital pain, nausea and vomiting. He felt that the headache was aggravated in the lying position and relieved in the upright or sitting position. Lumbar puncture revealed a high opening pressure of 20 cmH2O. Based on the symptoms and lumbar puncture pressure, RIH was considered. Meanwhile, with increasing CSF pressure, the Chiari-like tonsillar herniation disappeared. The patient was treated with acetazolamide and mannitol to reduce cranial pressure. At the 1-year follow-up, his headache was in remission.

## Discussion and conclusions

### Gorham-Stout disease

GSD is a rare disorder for which the aetiology is not fully understood [10]. The majority of patients presenting with GSD reported in the literature are children and young adults [2, 7, 8]. GSD is characterized by idiopathic and progressive bone resorption with replacement by lymphatic, vascular and fibrous tissue hyperplasia. The clinical manifestations and concomitant symptoms vary depending on the site of involvement. Although GSD is a benign disease, serious complications can occur, even life-threatening complications. When the spine and ribs are involved, including chylothorax or pleural effusion, the prognosis is suggested to be poor [4]. To date, there is no standard treatment. The main treatments reported in the literature are drugs, radiotherapy and surgery. Surgery is mainly for the treatment of complications [11].

### The association between GSD and CSF leakage

Patients with GSD may have CSF leakage. We reviewed the cases of GSD with CSF leakage (Table 1), which is more common in lesions involving the skull base [8, 9, 12–22], especially the temporal bone [8, 9, 12–15, 20–22]. CSF leakage may also occur when osteolytic lesions affect the spine [23–25]. We report the case of a male patient diagnosed with GSD with CSF leakage following an osteolytic lesion in the thoracolumbar spine. The patient initially had a diagnosis of sigmoid sinus stenosis. There is a certain relationship between venous sinus stenosis and idiopathic intracranial hypertension. Idiopathic intracranial hypertension is a well-known cause of skull base spontaneous CSF leakage, usually manifested as rhinorrhoea or otorrhea. However, in this case, the patient presented with orthostatic headache without rhinorrhoea, otorrhea, or visual disturbance. Moreover, skull base CT showed no skull base defect, and fundus examination showed no bilateral papilledema. Combined with the symptoms, signs and imaging examination, the CSF leakage in this patient was located in the spine rather than the skull base. The patient had undergone four spinal surgeries in the lower thoracic and lumbar regions due to massive osteolysis, so spinal MR images were difficult to obtain for this area. CT myelography was not performed to locate the leak due to the presence of cerebellar tonsillar herniation and concerns of additional leakage from a lumbar puncture. Therefore, the exact location of the CSF leak was not very clear. However, through the existing imaging methods, we inferred that the leakage point was located in the lower thoracic or lumbar segment and then located the repair site.

**Table 1:** Reported cases of GSD with CSF leakage

**Authors & Year**	**Age, Sex**	**Bone Involvement Causing CSF Leakage**	**Symptoms**	**Intracranial pressure**	**Rhinorrhoea**	**Chiari-like tonsillar herniation**	**Treatment of CSF leakage**	**Follow-up /outcome**	**Reference**
Present case	20, M	Thoracic and lumbar spine	Orthostatic headache	Low–high	None	Yes	EBP	1 year, rebound intracranial hypertension, improved	-
Yoshimoto et al. 2018	19, F	Femur and pelvis	Headache, motor weakness, visual acuity loss, papilledema	Low–high	None	Yes	EBP	1 year, improved	[7]
Morimoto et al. 2013	11, F	Right temporal bone (petrous apex)	Vertigo, headache, and pulsatile tinnitus, nausea, hearing loss	High	None	None	Surgical repair (extradural middle fossa approach with temporal fascia flap)	1 year, improved	[8]
Hosoya et al. 2020	25, M	Temporal bone	Otorrhea, meningitis	-	None	None	Infection control and bed rest	Improved	[12]
Aouad et al. 2022	3, F	Temporal bone	Fever, left otorrhea, swelling of the left neck	Low-rebound intracranial hypertension	None	Yes	Surgical treatment (mastoidectomy, fat graft)	Improved, rebound intracranial hypertension	[9]
Nagashima et al. 2017	25, F	Skull base (Right petrous apex)	Meningitis, headache, hearing loss	Normal	None	Yes	An endoscopic endonasal transsphenoidal approach	Follow-up	[13]
Cushing et al. 2010	12, M	Right petrous apex	Headache, nausea, vomiting, meningitis	Low	None	None	Middle ear and mastoid obliteration	6 months, Improved	[14]
Hernández-Marqués et al. 2011	2, M	Temporal bone	Fever, right otorrhea, vomiting, drowsiness, watery otorrhea, meningitis	-	None	None	Surgical interventions (mastectomy, placement of a patch, a lumbar drainage device)	The leakage ceased	[15]
Maroufi et al. 2022	11, M	Skull base	Neck pain	-	Yes	Cranial settling	Conservative management (bed rest, oral acetazolamide)	-	[16]
Fukayama et al. 2022	14, F	Skull base	Fever, neck pain, headache, vomiting, jaw pain, bacterial meningitis	-	None	None	Sirolimus	6 months, no recurrence of meningitis	[17]
Newland et al. 2008	27, M	Skull base	Chronic intermittent clear nasal discharge, headache	-	Yes	None	A sphenoid obliteration, lateral temporal bone resection, the Eustachian tube was obliterated	-	[18]
Nozawa et al. 2016	6, M	Skull base	CSF leakage, hearing loss, facial palsy	-	Yes	Herniation of cerebellar tissue into the internal auditory canal	Sirolimus	3 years, improved	[19]
Morinaga et al. 2022	33, F	The left temporal and sphenoid bones	Recurrent meningitis	-	None	None	Endoscopic endonasal surgery for dural reconstruction	12 months, improved	[20]
Watanabe et al. 2021	16, M	Temporal bone	Nasal discharge, right ear obstruction, fever, headache	Normal	Yes	None	Fistula closure by transmeatal approach	4 years, stable	[21]
Peragallo et al. 2018	18, F	The right temporal bone	Bacterial meningitis	Low–high	None	Yes	Zoledronic acid	Rebound intracranial hypertension	[22]
Yokoi et al. 2020	14, F	T9-10	Thoracic back pain, headache	Low	None	Yes	Dural repair, a blood patch	6 months, improved	[23]
Suero Molina et al. 2014	30, M	T11 vertebral body	Headache	Low	None	Yes	Neurosurgical management by repair of the dura	12 months, improved	[24]
Adler et al. 2011	7, F	Lumbar spine	Headache	Low	None	Yes	3 transforaminal blood patches	6 months, improved	[25]
Iyer et al. 1979	58, F	Skull (calvarial lesion)	Headache, vomiting, and delirium, meningitis	High	Yes	None	-	-	[26]

CSF leakage can be treated with conservative methods, EBP or surgical repair. For patients with mild CSF leakage, conservative management, such as strict bed rest, intravenous hydration and analgesics, can be chosen [6]. When conservative treatment is ineffective, blood patch therapy or surgical repair should be considered. In the reported cases of GSD with CSF leakage (Table 1), the authors generally chose surgical interventions for CSF leakage caused by skull base lesions [8, 9, 13–15, 18, 20, 21]. For CSF leakage caused by osteolysis of the spine, one patient underwent neurosurgical management to repair the dura [24]. Another patient underwent dural repair and a blood patch [23]. In addition, Adler et al. reported a GSD patient with CSF leakage involving the lumbar spine who underwent 3 transforaminal blood patches [25]. Considering that our patient had undergone four spinal surgeries, it would be more traumatic to perform another surgery to repair the tear causing CSF leakage. In addition, surgical repair carries risks and complications associated with recent spinal surgery. Under these circumstances, we performed lower thoracic EBP therapy. As we all know, EBP is a minimally invasive technique. It is performed by injecting a variable volume of autologous blood into the epidural space under local anaesthesia. EBP was initially used to treat postdural puncture headache. Currently, it is generally effective for CSF leaks caused by a variety of reasons, including dural weaknesses due to congenital or acquired factors and procedure-related CSF leaks [6]. The effect of EBP on CSF leak is mainly in two aspects. At earlier times, injection of autologous blood can immediately increase CSF pressure and improve the symptoms of intracranial hypotension. Subsequently, CSF leaks can be repaired by blood clot formation. EBP can be used as an intermediate step between conservative treatments and surgical measures. However, for patients with a history of spinal surgery, sometimes puncture is difficult due to their altered anatomy. EBPs can be performed under fluoroscopic, computed tomography or ultrasound guidance. It has also been reported that EBP can be successfully performed by changing the puncture space, placing an epidural catheter, or using a transforaminal approach [27, 28].

The aetiology of the development of CSF leakage in our patient remains unclear. Dural injury is the main cause of CSF leakage. We propose 2 explanations for the CSF leakage observed in this GSD patient. One reason may be progressive osteolysis involving the dura mater, resulting in CSF leakage, which was similar to that proposed in the previously reported literature [23, 24].

Alternatively, this patient had undergone multiple orthopaedic internal fixation operations in the past 10 years due to scoliosis caused by osteolysis. The last spinal surgery was revision and kyphosis osteotomy surgery involving the T10-L5 levels. The patient developed CSF leakage and intracranial hypotension syndrome six months after the last spinal surgery. This may be related to the weakening of the dura mater due to multiple revision surgeries. In spinal revision surgery, the anatomical structure of the surgical area is different from the normal structure, and the dura mater is widely adhered to the surrounding scar tissue, which increases the risk of dura mater breakage.

Intraoperative posterior pedicle screw placement and pedicle screw malposition may carry a potential risk of occult dural invasion with subsequent CSF leakage and intracranial hypotension. Any behaviour or condition that increases intra-abdominal pressure after surgery, such as cough, constipation, and sneezing, can lead to a transient increase in intradural pressure. CSF can rupture and tear the weak dura mater, resulting in the leakage of CSF. In this case, symptoms appeared half a year after the last surgery, suggesting that they might be secondary to a delayed dural leak. However, regardless of the above causes of CSF leakage, EBP therapy is effective.

### Rebound intracranial hypertension (RIH) after treatment of CSF leakage

It should be noted that after successful treatment of CSF leakage, RIH is sometimes encountered regardless of surgical repair methods or blood patch therapy [9, 29, 30]. In this case, the patient developed RIH 2 months after EBP therapy with a high opening pressure of 20 cmH2O. The pain metastasizes to the frontal or peri-orbital region and worsens in the supine position, accompanied by nausea and vomiting. According to the literature, symptoms of RIH can appear within hours to years after EBP therapy, with an incidence of 7% to 27.4% [29, 30]. A possible explanation for early RIH is a sharp increase in CSF pressure after the injection of autologous blood into the epidural space. The CSF volume returns to normal. However, compensatory mechanisms such as intracranial venous distension or subdural fluid collections are not immediately reversed [31]. Delayed RIH can occur weeks to even years after EBP treatment. In our case, RIH developed 2 months after EBP therapy. The potential mechanisms for delayed RIH include upregulation of CSF production and disrupted CSF reabsorption [31]. Patients with epidural effusion are more likely to develop RIH [30], as in our case. There are different opinions about the development of RIH after EBP treatment. Some authors consider it a complication [32], but others believe that the occurrence of RIH after EBP treatment is a good prognostic sign and a precursor of successful EBP treatment, which should not be misinterpreted as EBP treatment failure [30]. The management of RIH typically includes head elevation, the reduction of intracranial pressure with mannitol or glycerol and oral acetazolamide [30, 32]. Some patients with severe cases eventually require ventriculoperitoneal shunts to relieve their symptoms [9, 30]. Our patient was treated with acetazolamide and mannitol. At the 1-year follow-up, his headache was in remission.

### The association between CSF leakage and Chiari-like tonsillar herniation

Cerebellar tonsillar herniation can be secondary to chronic CSF leakage [5, 25]. In this case, we observed that the time between the onset of orthostatic headache and the diagnosis of CSF leakage was approximately 1 year. Early CSF leakage that is not treated in a timely manner may result in Chiari-like tonsillar herniation. When the volume of CSF is reduced, the buoyancy effect on the brain tissue is bound to decrease, with the occurrence of brain sagging and crowed posterior fossa. Cerebellar tonsillar herniation may be a manifestation of brain sagging. Conversely, with the increase in intracranial pressure after repair of the CSF leakage, the cerebellar tonsillar hernia can disappear. In our case, 2 months after EBP therapy, we observed increased intracranial pressure and the disappearance of tonsillar hernia. Regretfully, the patient was rechecked at a local hospital, so we were not able to obtain the imaging data of the postoperative re-examination. Dynamic CSF abnormalities may lead to reversible cerebellar tonsillar herniation [7]. Therefore, suboccipital decompression cannot be performed directly in accordance with Chiari I malformation when Chiari-like tonsillar herniation is suspected to be caused by CSF leakage. At this point, the key treatment is sealing a CSF leak through EBP therapy [5, 25]. Because of the potential risk of aggravating sagging of the cerebellar tonsils and additional CSF leakage, CT myelography was not performed for the patient. The exact lumbar puncture opening pressure and leak point could not be obtained before EBP therapy, which is a limitation of our article.

In summary, GSD is a rare disease. GSD involving the spine with CSF leakage is even more rare. For patients who have undergone multiple spinal surgeries, minimally invasive treatment of CSF leakage is an alternative treatment. CSF leakage is easily misdiagnosed. If early CSF leakage is not treated in a timely manner, cerebellar tonsillar herniation might occur. EBP can repair CSF leakage secondary to GSD and improve chronic brain sagging, with reversibility of Chiari-like malformations. It should be noted that after successful treatment of CSF leakage, RIH is sometimes encountered.

## Data Availability

The datasets used in this study are available from the corresponding author upon reasonable request.

## Abbreviations

GSD: Gorham-Stout disease
CSF: Cerebrospinal fluid
EBP: Epidural blood patch
RIH: Rebound intracranial hypertension
MR: Magnetic resonance
CT: Computed tomography
